# Polymorphisms and Expression Characteristics of the *ZSWIM7* Gene Are Associated with the Fertility of Male Allotetraploid of Red Crucian Carp × Common Carp

**DOI:** 10.3390/ani16020352

**Published:** 2026-01-22

**Authors:** Tao Dai, Minglin Dong, Siyang He, Weiling Qin, Conghui Yang, Yi Zhou

**Affiliations:** Engineering Research Center of Polyploid Fish Reproduction and Breeding of the State Education Ministry, College of Life Sciences, Hunan Normal University, Changsha 410081, China; daitao2000311@163.com (T.D.); minglin2000@foxmail.com (M.D.); hesiyang1015@163.com (S.H.); weiling_qin@163.com (W.Q.)

**Keywords:** *ZSWIM7*, allotetraploid, broodstock development, genomic selection, testis, single nucleotide polymorphism (SNP)

## Abstract

Allotetraploid fish produced by distant hybridization are valuable germplasm for the mass production of sterile triploids. The allotetraploid crucian–carp hybrid (4nAT, 4n = 200) is derived from the intergeneric cross between a female red crucian carp (*Carassius auratus* red var., 2n = 100) and a male common carp (*Cyprinus carpio* L., 2n = 100). However, after 33 successive generations, this lineage faces a critical bottleneck in maintaining male fertility. The present study aimed to develop new biomarkers for testicular development and characterize the associated functional gene expression profile in 4nAT. Following whole-genome resequencing and selection signature analysis of 15 male 4nAT individuals from each of the high-development group (HDG) and low-development group (LDG), *ZSWIM7* (Zinc Finger SWIM-Type Containing 7), a gene implicated in reproductive development, was selected as a candidate for further fertility association studies. Seven SNPs were screened in the coding region of *ZSWIM7* of 70 4nAT males; among these, SNP3 (c.23T/C) exhibited a significant correlation between genotypes and testicular development: individuals with the CT genotype showed a higher gonadosomatic index (1.17 ± 0.68 vs. 0.65 ± 0.50) and greater counts of mature spermatozoa (2537.67 ± 283.95 vs. 341.56 ± 121.66) compared to those with the TT genotype. Further quantitative PCR and immunofluorescence assays demonstrated that *ZSWIM7* was highly expressed in the testis and specifically localized to the nuclei of early meiotic primary spermatocytes. Collectively, these results establish *ZSWIM7* as a promising biomarker for 4nAT testicular development, offering a potential molecular tool for maintaining male fertility in allotetraploid fish breeding.

## Simple Summary

The allotetraploid crucian–carp hybrid is a valuable germplasm because it can be used to produce sterile triploids with superior growth performance. However, maintaining male fertility has become a challenge after many generations. In this study, based on genome resequencing, we focused on the *ZSWIM7* gene and discovered an SNP molecular marker associated with better testis development and high mature sperm counts. Further spatiotemporal expression analysis demonstrated that *ZSWIM7* is mainly active in the testis, specifically in early meiotic primary spermatocytes. Our findings highlight the potential of *ZSWIM7* as a candidate gene for marker-assisted selection to maintain male fertility in allotetraploid breeding.

## 1. Introduction

Maintenance of the reproductive capacity in cultured fish is a fundamental safeguard for the continuous production of high-quality fry [1,2]. In aquaculture, inbreeding depression, poor management practices, and other factors can lead to a decline in fertility [3]. This decline directly affects reproductive efficiency and seedling yield and may result in genetic degeneration and reduced population adaptability, ultimately hampering the long-term sustainability of the industry [4]. Therefore, implementing effective management strategies and breeding programs to maintain male fertility and ensure the continued success and sustainability of aquaculture operations is critical.

The allotetraploid crucian–carp lineage (4nAT, 4n = 200) was derived from intergeneric hybridization of crucian carp (*Carassius auratus* red var., ♀, 2n = 100) × common carp (*Cyprinus carpio* L., ♂, 2n = 100) [5]. As a fertile (in both sexes) and landmark vertebrate allotetraploid lineage obtained through distant hybridization, 4nAT reproduces hybrid polyploidization events in fish and provides an important model for studying species evolution [6]. In addition, hybridizing 4nAT with diploid crucian or common carp produces sterile triploids that exhibit superior growth performance and are now widely farmed in China [7,8,9]. This lineage, established in the 1990s, has persisted for 33 consecutive generations. After years of breeding, some individuals have shown inevitable declines in reproductive capacity due to inbreeding depression. Therefore, selecting highly fertile individuals is important to ensure reproductive capacity and sustain this unique germplasm resource.

Sperm quality plays an important role in the preservation and sustainable utilization of germplasm resources [10]. High-quality sperm is essential for ensuring successful fertilization, proper embryonic development, and stable inheritance of desirable genetic traits [11]. In aquaculture, sperm quality directly influences the efficiency of seed production and the sustainability of long-term breeding programs [12]. Therefore, the healthy development of the testis is fundamental to maintaining fish germplasm resources, particularly the valuable 4nAT lineage. Given that traditional morphological assessment of testicular development compromises fish survival, utilizing molecular markers to identify individuals with high fertility potential presents a promising alternative [13,14]. Studies of molecular markers associated with testicular development and sperm quality have been conducted in several fish species, including the tiger pufferfish (*Takifugu rubripes*) [15] and brook charr (*Salvelinus fontinalis*) [16], which not only significantly improve our understanding of reproductive mechanisms but also aid in selective breeding.

Genomic resequencing is a powerful approach to identify single-nucleotide polymorphisms (SNPs) [17], and by correlating these polymorphic sites with reproductive traits, molecular markers for fertility benefits can be screened [18]. To date, no fertility-related markers have been established for 4nAT lineage, which hinders their effective conservation and breeding programs. In the present study, we first utilized genome resequencing data from different 4nAT populations to identify *ZSWIM7* (Zinc finger SWIM-Type containing 7) as a candidate gene. Subsequently, we identified an SNP in the coding region of *ZSWIM7* that was significantly correlated with testis development and further investigated the gene’s spatiotemporal expression characteristics during meiosis. These findings provide valuable SNP markers for selecting male 4nAT lineages for reproduction and breeding.

## 2. Materials and Methods

### 2.1. Experimental Animals

Fish used in this study were collected from the Fish Genetic Breeding Center of Hunan Province (Changsha, China). The fish were fed in a 667 m^2^ pond and fed twice a day (8:00 and 17:00) for more than one year, and a total of 100 individuals of male 4nAT were randomly selected for the study in December 2023. Before the experiment, fish were anesthetized with tricaine methanesulfonate (MS-222, Sigma–Aldrich, Shanghai, China) dissolved in distilled water at a concentration of 80 mg/L. All the experiments were conducted following the instructions of the Administration of Affairs Concerning Experimental Animals for the Science and Technology Bureau of China and approved by the Animal Care Committee of Hunan Normal University, Changsha, China (ethics permit number: 2020-014).

### 2.2. Phenotypic Data Collection

Thirty male 4nAT individuals were randomly selected for morphological measurement using an automated phenotyping system. Fillet weight (FW) was determined by weighing the dorsal musculature dissected from both sides of the spine after descaling. Based on initial morphological data, the following core developmental indicators were selected for evaluation: body weight (BW), Fulton’s condition factor (K = BW/SL^3^ × 100), head length/standard length ratio (HL/SL), caudal peduncle depth/caudal peduncle length ratio (CPD/CPL), and fillet yield (FY = FW/BW × 100%). To eliminate scale differences, each indicator was standardized using Z-scores, and a composite developmental score (CDS) was calculated as the sum of the Z-scores for BW, K, HL/SL, CPD/CPL, and FY. According to the CDS rankings, the 15 individuals with higher scores were assigned to a high-development group (HDG), and the remaining 15 individuals were assigned to a low-development group (LDG). Inter-group differences were determined by an independent samples *t*-test using SPSS 27.0 software, and the distribution of each indicator was visualized using scatter plots.

### 2.3. Whole-Genome Resequencing and Bioinformatics Analysis

Genomic DNA was extracted from tail fins collected from thirty 4nAT individuals using the standard phenol-chloroform method. The quantity and integrity of DNA were examined by an Agilent 2100 Bioanalyzer (Agilent, Santa Clara, CA, USA) and a Qubit 4.0 (Invitrogen, Waltham, MA, USA). DNA libraries were constructed using Illumina’s TruSeq DNA PCR-Free Library Prep Kit and subjected to paired-end sequencing on an Illumina NovaSeq 6000 platform (Illumina, San Diego, CA, USA). Raw sequencing data were processed using the Illumina bcl-convert software (v3.9.5) for demultiplexing and FASTQ file generation.

Raw data were processed using a standard quality control tool for second-generation sequencing data (Trimmomatic, v0.39) to remove low-quality reads. To improve SNP calling and gene mining, both the goldfish genome (NCBI accession number: GCF_003368295.1) and a combined genome of goldfish and common carp (NCBI accession number: GCF_018340385.1) were used as a reference genome. Sequence alignment was performed using BWA-MEM (v0.7.17), and duplicate reads were marked using Picard MarkDuplicates of GATK (v4.2.0.0) [19]. Joint variant calling was conducted using the HaplotypeCaller of GATK (v4.2.0.0) to generate the gVCF files [20]. In the variant discovery analysis using GATK, we set the *--sample-ploidy* parameter differently to reflect the ploidy assumption for each reference genome: to 2 or 4 when using the goldfish genome as reference, and to 2 when using the combined genome. The high-quality SNP dataset was obtained using VariantFiltration of GATK (v4.2.0.0) with the following criteria: QUAL < 30.0, QD < 2.0, MQ < 40.0, FS > 60.0, SOR > 3.0, MQRankSum < −12.5, and ReadPosRankSum < −8.0. Distribution of SNPs across chromosomes was visualized in R software (v4.3.3) using the CMplot package (v4.51). Individual genotypes were parsed using BCFtools (v1.17) to calculate homozygosity rates, heterozygosity rates, and missing rates. Population genetic analyses were conducted using PLINK (v1.9), including SNP density (SNP/kb), observed heterozygosity (Ho), minor allele frequency (MAF), polymorphism information content (PIC), and nucleotide diversity (π).

Artificial selection signals were detected across the genome using Weir and Cockerham’s fixation index (FST) method [21] and the cross-population composite likelihood ratio (XP-CLR) method [22]. FST analysis was performed using VCF tools (v0.1.15), which calculate genetic differentiation indices between populations based on a hierarchical analysis of variance model [23]. Considering the heterozygous nature of 4nAT lineage, only autosomal regions were retained in the analysis, and low-quality sites were filtered (MAF < 0.01, missing rate > 10%). The XP-CLR method identifies selection signals by comparing allele frequency differences between populations while incorporating linkage disequilibrium (LD) information, allowing the detection of polygenic co-evolution events. XP-CLR analysis was conducted using software released by the Reich Lab at Harvard University (version 1.0, available at https://reich.hms.harvard.edu/index.php/software, accessed on 4 August 2023). The parameters were set as follows: a maximum SNP spacing of 200 kb, an allele frequency difference threshold of 0.8, and a default Gaussian kernel for the likelihood-weighting model. To standardize the analysis scale, the genome was divided into non-overlapping 50 kb windows with a step size of 25 kb (50% overlap between adjacent windows), and each window was required to contain at least 10 valid SNPs (low-density windows were filtered to reduce random noise). Significant selection signals were defined as genomic windows with FST or XP-CLR scores in the top 1% of the distribution when using the diploid goldfish reference genome (ploidy = 2 in GATK). In contrast, a top 5% threshold was applied when using either the tetraploid goldfish reference (ploidy = 4 in GATK) or the diploid combined reference genome (ploidy = 2 in GATK). Overlapping regions detected by both methods were intersected using the intersect module of Bedtools (v2.30.0) [24], and genome-wide selection signals were visualized using Manhattan plots (ggplot2, v3.5.2).

GO and KEGG functional annotation and pathway enrichment analyses were performed using the clusterProfiler package (v4.10.0) in R software (v4.3.3) to further investigate the biological functions and pathway characteristics of the candidate genes. The significance of gene set enrichment in GO categories was assessed using the hypergeometric test, and the Benjamini–Hochberg (BH) method was applied to correct for the false discovery rate (FDR < 0.05) caused by multiple-hypothesis testing. KEGG pathway enrichment analysis was conducted using the KEGG Orthology database to identify key signal transduction pathways related to reproductive development (*p* < 0.05). Significantly enriched GO terms and KEGG pathways were visualized using the ggplot2 package (v3.5.2) in R software (v4.3.3), highlighting their hierarchical relationships and core functional modules. Finally, entries meeting the criteria (GO: FDR < 0.05; KEGG: *p* < 0.05) were interpreted biologically, focusing on functional categories associated with regulating reproductive development in 4nAT lineage.

### 2.4. Preliminary Screening and Genotyping of SNPs in the ZSWIM7 Gene Coding Region

After identifying the *ZSWIM7* gene as a potential marker for reproductivity, we collected 70 males for screening and genotyping of SNPs. After DNA extraction, the sequences of the *ZSWIM7* gene were amplified by PCR. The specific primers were designed based on the ZSWIM7 cDNA sequence (ENSCARG0000000557) of goldfish (Table S19). The PCR conditions were 94 °C for 3 min for initial denaturation, 35 cycles of 94 °C for 30 s, 52 °C for 30 s, and 72 °C for 2 min, and a final extension at 72 °C for 5 min. PCR products were purified using a DNA purification kit (Sangon, Shanghai, China) and sequenced by Sangon Biotech (Shanghai) Co., Ltd.

Multiple sequence alignment of the sequencing data was performed using SnapGene (v8.0). Sites showing base differences at the same genomic coordinates with coverage in >30% of the samples were defined as SNPs. Based on HaploView (v4.2) [25], the minor allele frequency (MAF ≥ 0.05) was calculated for each variant site to screen for valid polymorphic loci that meet population genetics criteria. The .abi files generated from the sequencing and electropherogram signals were jointly analyzed using SnapGene (v8.0) and Chromas (v2.6.6). A single peak was interpreted as a homozygous locus, whereas overlapping double peaks indicated a heterozygous genetic variation.

After obtaining the genotyping data, the genetic parameters of the SNP loci were calculated using HaploView (v4.2) software. These parameters included genotype frequency, allele frequency, expected heterozygosity (He), effective number of alleles (Ne), and PIC.

### 2.5. Linkage Disequilibrium Assays and Diplotype Analysis

Linkage disequilibrium (LD) analysis of polymorphic loci was conducted using HaploView (v4.2) software [25]. Haplotype blocks were constructed based on linkage patterns among SNPs, and haplotype frequencies were statistically estimated. Low-frequency haplotypes with a frequency threshold < 0.05 were excluded from the analysis. D’ and r^2^ values were used as key metrics to quantify the degree of linkage: r^2^ ranged from 0 to 1, with higher values indicating a stronger linkage between the loci. When both D’ and r^2^ reach a theoretical maximum of 1, a complete link between the alleles is suggested. In addition, diplotypes formed by combinations of haplotypes with frequencies > 0.03 were included in subsequent analyses.

### 2.6. Correlation Between SNPs of ZSWIM7 Gene Coding Region and Reproductivity

The reproductivity of male 4nAT was determined using the gonadosomatic index (GSI) and the relative proportions of germ cell types. GSI was calculated using the following formula: GSI = (Gonad weight/Body weight) × 100. The proportions of germ cell types were analyzed using paraffin sections. Testicular tissues were embedded in paraffin and stained with hematoxylin and eosin (H&E) for histological examination. Paraffin sections were observed under a light microscope (Zeiss, Oberkochen, Germany), and 10 images were captured per section. Applying a machine learning-based approach, spermatocytes and spermatids within these images were quantified using the “Trainable Weka Segmentation” plugin in ImageJ software (v1.54). The relative proportions were calculated using previously described methods. The gonad weight, body weight, GSI, and proportion of spermatocytes and spermatids from different SNP genotypes were analyzed by *t*-test and one-way analysis of variance (ANOVA) using IBM SPSS Statistics (v27). Significant differences between groups were confirmed at *p* < 0.05. All the data were shown as average ± standard deviation.

### 2.7. Quantitative PCR (qPCR)

To evaluate the mRNA expression levels of the *ZSWIM7* gene using qPCR, we collected multiple tissues, including heart, brain, spleen, liver, kidney, foregut, midgut, hindgut, muscle, gill, and testis, from 4nAT (*n* = 3 per tissue). Total RNA was isolated using TRIzol (Invitrogen, Waltham, MA, USA), and cDNAs were prepared using the PrimeScript^TM^ RT reagent Kit (Perfect Real Time) (Takara, Kusatsu, Japan). qPCR was performed using QuantStudio 3 (Thermo Fisher Scientific, Waltham, MA, USA). The reaction conditions were initial denaturation at 95 °C for 10 min, followed by 40 cycles including 95 °C for 15 s and 60 °C for 1 min. Primers used were listed in Table S19. After amplification, melt-curve analysis was performed to determine whether the amplification was specific. The relative expression was calculated by the 2^−ΔΔCt^ method [26]. Significant differences among the groups were determined by one-way ANOVA using IBM SPSS Statistics (v27). Significant differences among the groups were confirmed at *p* < 0.05.

### 2.8. Immunofluorescence Assay

ZSWIM7 and PCNA protein expression were detected in the testes of 4nAT using an immunofluorescence assay. Testicular tissues were fixed in 4% paraformaldehyde, embedded in OCT compound (Sakura, Osaka, Japan), and sectioned into frozen slices for immunofluorescence analysis. The frozen sections were permeabilized with 0.1% Triton X-100 (200 μL) for 15 min and three washes with TBST. Non-specific binding was blocked using 3% bovine serum albumin (BSA; Sigma, St. Louis, MO, USA) at room temperature for 30 min, and sections were then rinsed once with 500 μL TBST. Primary antibodies were applied by incubating the sections overnight at 4 °C with 200 μL of the following antibodies diluted in TBST: ZSWIM7 (1:200, HPA054681, Sigma), VASA (1:200, ab209710, Abcam, Cambridge, UK), Cyclin A (1:200, SAB4503499, Sigma), PCNA (1:200, P8825, Sigma), and SOX9 (1:100, ab202516, Abcam). After three additional TBST washes (500 μL each), the sections were incubated with 200 μL of the appropriate Alexa Fluor-conjugated secondary antibodies—either goat anti-rabbit (A-11008, Thermo Fisher Scientific, Waltham, MA, USA) or goat anti-mouse (A-11001, 1:1000, Thermo Fisher Scientific, Waltham, MA, USA)—followed by another three washes with TBST. Subsequently, the nuclei were counterstained with DAPI (1:500 dilution in PBS; D9542, Sigma) for 5 min and then rinsed once with TBST. Fluorescent signals were visualized using a BX63 automated fluorescence microscope (Olympus, Tokyo, Japan).

## 3. Results

### 3.1. Phenotypic Measurement and Population Division

**Figure 1:**
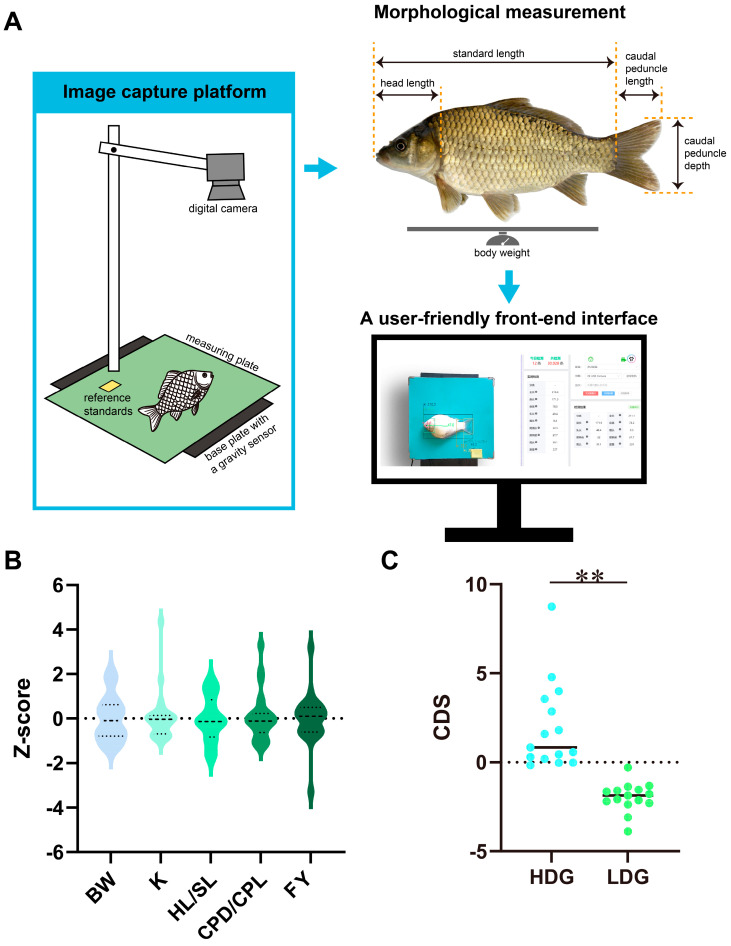
Morphological indicators and developmental composite score of thirty male 4nAT individuals. (**A**) An automated system for morphological measurement of 4nAT individuals. (**B**) The distribution of the standardized values of morphological indicators. BW: body weight; K: Fulton’s condition factor; HL/SL: head length/standard length ratio; CPD/CPL: caudal peduncle depth/caudal peduncle length ratio; FY: fillet yield. The vertical axis represents Z-score normalized values to eliminate dimensional differences. (**C**) Inter-group differences in comprehensive developmental score (CDS). HDG: high-development group (*n* = 15); LDG: low-development group (*n* = 15). ** indicates an extremely significant difference in CDS between groups (*p* < 0.01).

Morphological measurement was performed using an automated phenotyping system (Figure 1A and Figure S1, and Table S1). The Z-score standardized values of the five developmental indicators (BW, K, HL/SL, CPD/CPL, and FY) approximated a normal distribution, indicating that the samples are suitable for phenotypic and genomic resequencing analyses (Figure 1B). Based on the CDS ranking, which is the sum of the Z-scores for the five indicators, the thirty 4nAT individuals were stratified into two groups: a high-development group (HDG, *n* = 15) and a low-development group (LDG, *n* = 15). An independent samples *t*-test revealed a highly significant difference in CDS between the HDG and LDG (*p* < 0.01, Figure 1C), confirming that the population stratification strategy could effectively support subsequent selection signature detection analysis.

### 3.2. Detection of Polymorphic Sites by Genome Resequencing

Based on the diploid goldfish reference genome (ploidy = 2 in GATK), a total of 52.01 million SNPs were identified from the whole-genome resequencing data, and 26.07 million of these met the filtering criteria. The average SNP density (14.371 SNPs/Kb), observed heterozygosity (Ho = 0.4988 ± 0.2614), minor allele frequency (MAF = 0.2955 ± 0.1435), and polymorphic information content (PIC = 0.3463 ± 0.1475) collectively indicate a high level of genomic variation in the 4nAT lineage and demonstrate that these SNPs are informative for genetic analyses. Among the detected SNP loci, the average number of sites where both alleles in each sample were type-A (AA genotype) was 212.9 ± 97.8 million, accounting for an average of 40.94% ± 1.8% of all detected loci. The average number of sites where both alleles were type-B (BB genotype) was 64.6 ± 18.2 million, representing 12.42% ± 0.3% of all loci (Table S5). The average total homozygosity was 53.36%, whereas the average heterozygosity was 33.20%, consistent with the characteristics of hybrid vigor in tetraploids. The number of high-quality SNPs per chromosome ranged from 1,514,797 (Chr7) to 68,182 (Chr33), with an average of 836,583 high-quality SNPs per chromosome. The highest SNP density regions contained more than 59,968 SNPs/Mb, whereas the lowest density regions contained only 1 SNP/Mb (Figure S2). We also analyzed SNPs using a tetraploid goldfish reference (ploidy = 4) and a diploid combined reference (ploidy = 2); however, the SNP yield and density from these analyses were markedly lower than those from the diploid goldfish reference (ploidy = 2) analysis, likely attributed to reduced sequencing depth (Figures S3 and S4, Tables S6 and S7).

**Figure 2:**
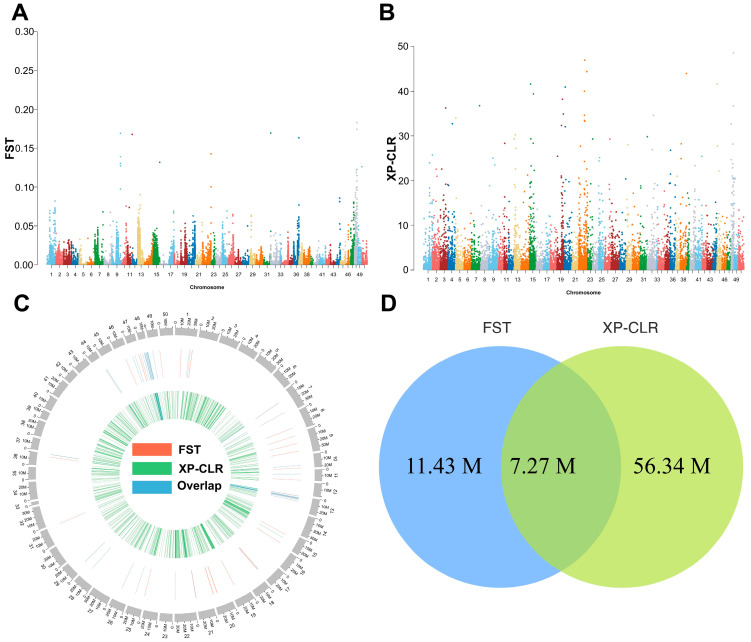
Identification of high-frequency mutation regions in the 4nAT genome by FST and XP-CLR analysis. (**A**) Manhattan plot of FST values (calculated in 50 kb windows with a 25 kb step size), with the *y*-axis representing FST and the *x*-axis indicating chromosomes. (**B**) Manhattan plot of XP-CLR scores (calculated in 50 kb windows with a 25 kb step size), with the *y*-axis representing XP-CLR and the *x*-axis indicating chromosomes. (**C**) Circos plot showing genomic distribution of selective regions detected independently by FST (red), XP-CLR (green), or both methods (blue). (**D**) Venn diagram illustrating the overlap in selective region sizes captured by FST and XP-CLR methods.

**Figure 3:**
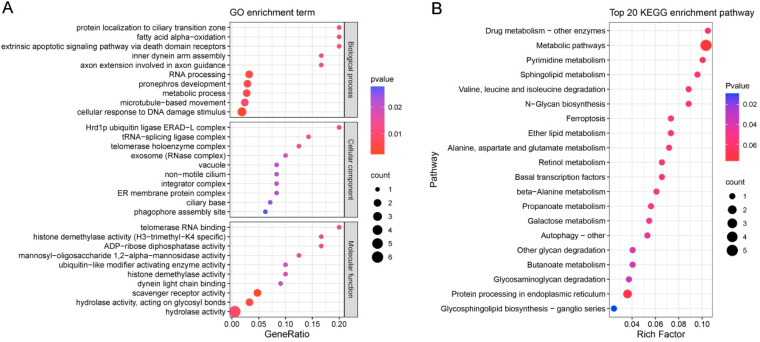
GO (**A**) and KEGG (**B**) enrichment analysis of the functional genes in high-frequency mutation regions.

This study used the FST and XP-CLR methods to analyze the genomic features of 4nAT lineage under artificial selection pressure. Using the diploid goldfish reference genome (ploidy = 2 in GATK) and a top 1% significance threshold, the FST method identified 11.14 Mb of significantly differentiated regions across the genome (Figure 2A,C), encompassing 489 functional genes. These regions may reflect the differences in allele frequencies resulting from long-term selection. The XP-CLR method, which is based on the continuity pattern of allele frequencies, detected selection signal regions totaling 56.34 Mb (Figure 2B,C), including 5051 genes, suggesting that these loci may have been under positive selection or driven by adaptive evolution. Notably, there was a significant overlap between the two methods; a candidate selection region of 7.27 Mb and its 183 included genes were identified using both algorithms (Figure 2D). GO enrichment analysis indicated that the most enriched GO terms were cell movement (inner dynein arm assembly and microtubule-based movement) and metabolism (fatty acid alpha oxidation and metabolic process) (Figure 3A). KEGG analysis showed that pathways involved in metabolism and protein synthesis were enriched (Figure 3B). Based on the enrichment analysis results, we systematically identified key genes involved in biological processes and signaling pathways related to reproductive development in 4nAT lineage; 21 significantly associated candidate genes were identified (Table S15). Among them, *ZSWIM7* is functionally associated with the activation of multiple repair mechanisms during the cellular response to DNA damage-induced stress (GO:0006974) and plays a role in the high-fidelity repair of double-strand breaks via the homologous recombination pathway (GO:0000724). Importantly, this gene was located within the top 6% selected regions of the XP-CLR analysis under the tetraploid goldfish reference (ploidy = 4 in GATK), as well as in the overlapping top 5% regions of both FST and XP-CLR analyses under the diploid combined reference genome of goldfish and common carp (ploidy = 2 in GATK) (Figures S5 and S6, Tables S16–S18). This consistent signal confirms significant genetic differentiation at this locus between the HDG and LDG. Thus, our subsequent study focused on *ZSWIM7* to investigate the association between its polymorphisms and gonadal development in 4nAT lineage.

### 3.3. Genotyping and Genetic Diversity Analysis of SNPs in the Population

**Figure 4:**
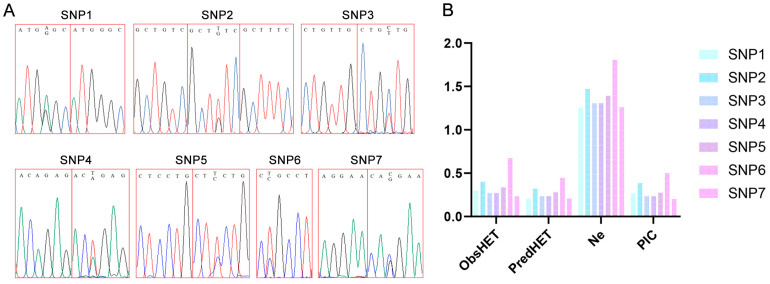
Screening of SNP loci and genotyping profiles in the *ZSWIM7* coding region. (**A**) SNPs were checked through the AB1. (**B**) Population genetic polymorphism analysis of the *ZSWIM7* gene.

Seven SNPs were screened by *ZSWIM7* sequencing (Figure 4A): SNP1 (c.4G/A), SNP2 (c.18G/T), SNP3 (c.23T/C), SNP4 (c.50A/T), SNP5 (c.318C/T), SNP6 (all heterozygous c.324T/C), and SNP7 (c.360G/C). Among these, SNP1, SNP2, SNP3, SNP4, SNP5, and SNP7 exhibited two genotypes, whereas SNP6 was heterozygous (Table S20). The genotype and allele frequencies of seven SNP loci in *ZSWIM7* were analyzed. The genotypes with the highest frequency in SNP loci were SNP1-GG (68.57%), SNP2-GG (60%), SNP3-TT (72.86%), SNP4-AA (72.86%), SNP5-CC (67.14%), SNP6-TC (100%), and SNP7-GG (77.14%), with the highest frequency alleles of SNP1-G (84.28%), SNP2-G (73.57%), SNP3-T (86.43%), SNP4-A (86.43%), SNP5-C (83.57%), SNP6-T=C (50%), and SNP7-G (88.57%), respectively. The analysis data of genetic parameters of SNP loci (Ho, He, Ne, and PIC) were analyzed (Figure 4B). He ranged from 0.202 to 0.446. SNP1, SNP2, SNP5, and SNP6 were moderately polymorphic, with PIC ranging from 0.25 to 0.5. SNP3, SNP4, and SNP7 showed low polymorphism, with PIC values < 0.25.

### 3.4. Correlation Analysis Between Genotypes and Phenotypes

**Figure 5:**
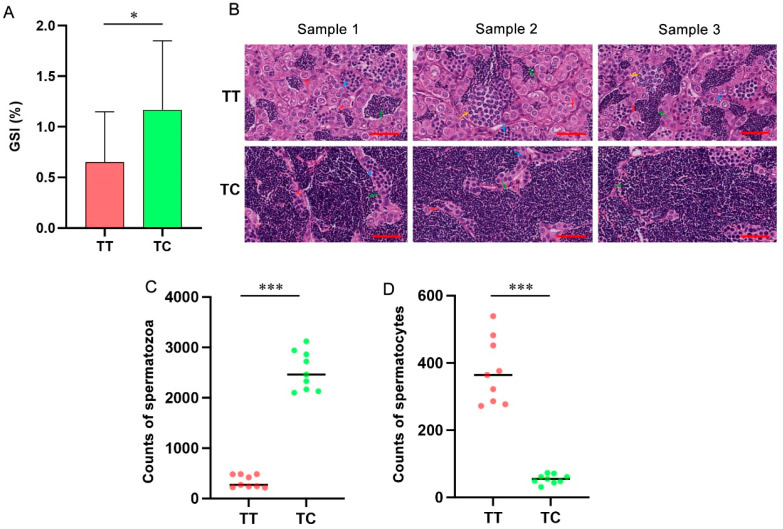
Correlation analysis between SNP3 genotypes and testicular development. (**A**) GSI of the TT and TC genotype groups. *, *p* < 0.05. (**B**) Histological analysis of the testes of the TT and TC genotype groups. The arrows denote different cell types: red for spermatogonia, blue and yellow for spermatocytes, and green for spermatozoa. Scale bar = 50 μm. (**C**) Spermatozoa count of the testes of the TT and TC genotype groups. ***, *p* < 0.001. (**D**) Spermatocytes count of the testes of the TT and TC genotype groups. ***, *p* < 0.001.

The correlation between different genotypes and the testicular development of 4nAT was analyzed. Only SNP3 (c.23T/C) showed significant differences in the gonad weight, body weight, and GSI among the different genotypes (Table S21). The SNP with CT genotype had 1.17 ± 0.68 GSI, which was significantly higher than the TT genotype with the GSI as 0.65 ± 0.50 (*p* < 0.05) (Figure 5A). Subsequently, we analyzed the correlation between testicular histological characteristics and the observed genotypes. In the TC group, the seminiferous tubules were predominantly occupied by mature spermatozoa. In contrast, in the TT group, most germ cells were observed at the spermatocyte stage, reflecting an earlier stage of spermatogenic development (Figure 5B). The average counts of spermatids were significantly higher in the TC group than those in the TT group (2537.67 ± 283.95 vs. 341.56 ± 121.66) (*p* < 0.05). In contrast, the average counts of spermatocytes were significantly lower in the TC group than those in the TT group (54.78 ± 13.39 vs. 374.45 ± 97.04) (*p* < 0.05) (Figure 5C,D).

### 3.5. Linkage Disequilibrium (LD) Analysis and Haplotype Construction

**Figure 6:**
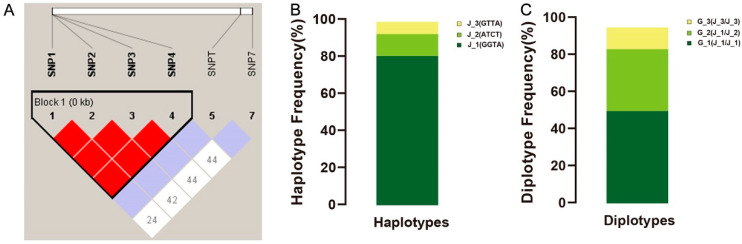
Linkage disequilibrium analysis and haplotype construction. (**A**) Linkage disequilibrium analysis by HaploView. Red color means strong LD and the block box marks the LD SNPs. (**B**) Frequency distribution of haplotypes. (**C**) Frequency distribution of diplotypes.

LD analysis and haplotype constructions were performed. A haplotype Block 1 was found constructed by SNP1, SNP2, SNP3, and SNP4 by LD analysis (D’ > 0.75, r^2^ > 0.33) (Figure 6A). Based on the linkage disequilibrium analysis of 70 4nAT, three major haplotype blocks were identified—J_1_ (GGTA), J_2_ (ATCT), and J_3_ (GTTA)—with corresponding frequencies of 80.0%, 11.4%, and 6.4%, respectively (Figure 6B). Three genotypes were identified with estimated frequencies of 48.57% for G_1_ (J_1_/J_1_), 32.86% for G_2_ (J_1_/J_2_), and 12.86% for G_3_ (J_3_/J_3_) (Figure 6C). The analysis revealed a significant correlation between the diplotypes of the *ZSWIM7* gene and GSI in the 4nAT lineage. Diplotype distributions were as follows: G_1_ (*n* = 39), G_2_ (*n* = 23), and G_3_ (*n* = 9). Notably, individuals with diplotype G_3_ exhibited a significantly higher GSI (1.21 ± 0.34) than those with G_1_ or G_2_ (Table S22).

### 3.6. ZSWIM7 Expression Characteristics

**Figure 7:**
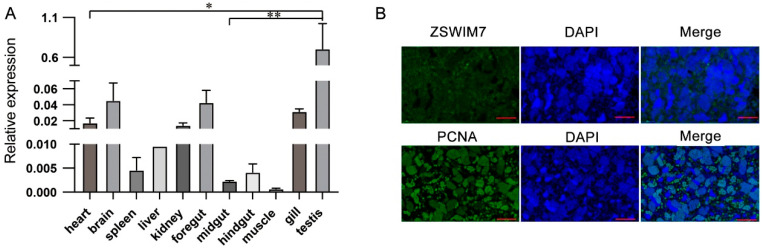
Gene expression of *ZSWIM7*. (**A**) Tissue distribution of *ZSWIM7* in allotetraploid. *, *p* < 0.05; **, *p* < 0.01. (**B**) Immunofluorescence analysis of ZSWIM7 and PCNA. Scale bar = 40 μm.

qPCR showed that *ZSWIM7* was present in all tested tissues. Among the tissues, the testes showed the highest expression of *ZSWIM7* compared to other tissues (*p* < 0.05) (Figure 7A). In the nuclei of the primary spermatocytes of the 4nAT lineage, the green, fluorescent signal of *ZSWIM7* exhibited a punctate (foci-like) distribution (Figure 7B). This localization pattern is similar to that of PCNA (a nuclear proliferation marker) (Figure 7B). This characteristic aligns with the role of *ZSWIM7* in meiotic DNA damage repair. Although PCNA is broadly distributed in proliferating spermatogonia and spermatocytes, ZSWIM7 is specifically concentrated in the nuclei of early meiotic primary spermatocytes. In contrast, ZSWIM7 signals were completely absent in spermatids because of their highly condensed chromatin and cessation of DNA repair activity.

## 4. Discussion

The conservation of allotetraploid germplasm resources, such as the 4nAT lineage, is crucial for producing triploid fish and for evolutionary, genetic, and breeding studies [27,28]. Nevertheless, the lack of molecular markers for gonadal development phenotypes results in wasteful consumption of parental breeding stocks and hinders breeding efficiency. Here, based on whole-genome resequencing data and selection signature detection analysis, the present study selected *ZSWIM7* as a candidate gene, identified SNP loci significantly associated with testicular development, and further investigated the gene’s spatiotemporal expression characteristics. This work provides fundamental data for 4nAT conservation and advances our understanding of *ZSWIM7* in testicular development.

To identify genomic regions related to the growth situation of 4nAT, we employed genome resequencing combined with FST and XP-CLR analysis to scan population selection signatures [29,30]. A comparative analysis revealed a 7.27 Mb genomic region with high genetic variation in the 4nAT genome, which was consistently identified by both methods. Within this region, 183 functional genes were annotated, which GO terms and KEGG pathway analyses showed were enriched in functions related to cell movement, metabolism, and protein synthesis. Cell movement is crucial for spermatogenesis, enabling germ cell migration and proper positioning within the seminiferous epithelium [31] and facilitating the structural remodeling during spermatid maturation, which includes cytoskeletal reorganization and flagellar formation [32]. During spermatogenesis, protein synthesis serves as a core supportive mechanism, facilitating the rapid cell division, differentiation, and structural remodeling necessary for germ cell development through the timely production of proteins involved in key events like meiosis, chromatin remodeling, acrosome formation, and flagellum assembly [33,34]. Therefore, the regions with a high SNP density within these functional genes could be candidate molecular markers related to reproductive development.

Male 4nCT are the parents used for producing the high-quality triploid fish “Xiangyun crucian carp No. 2”. Both the growth status and gonad development of male 4nCT are key biological characteristics of practical concern in our aquaculture production. Therefore, from the candidate genes identified via genomic differentiation associated with growth, we subjectively selected a reproductive development-related gene for fertility association studies. Considering the polyploid nature of 4nCT and to eliminate reference genome bias, two reference genomes and three SNP calling strategies were employed in this study: Strategy 1—the goldfish reference genome with diploid SNP calling (set --sample-ploidy = 2 in GATK); Strategy 2—the goldfish reference genome with tetraploid SNP calling (set --sample-ploidy = 4 in GATK); Strategy 3—a combined goldfish–common carp reference genome with diploid SNP calling (set --sample-ploidy = 2 in GATK). Furthermore, we performed selection signal analyses (FST and XP-CLR) based on SNPs obtained from all three strategies. When applying a top 1% threshold, we found no overlap among the candidate genomic regions identified by the different strategies, which may be attributed to the limited sample size and sequencing depth in this study. However, crucially, we found that the *ZSWIM7* gene, initially identified using the diploid goldfish reference, also appeared in the top 6% significance region of the XP-CLR analysis based on the tetraploid goldfish reference genome (GATK ploidy = 4), and the overlap region with the top 5% significance from both FST and XP-CLR analyses based on the combined goldfish–common carp reference genome (GATK ploidy = 2). This consistent signal across multiple analytical frameworks strongly indicates that the genomic region containing *ZSWIM7* exhibits clear differentiation between the HDG and LDG, marking it as a functionally relevant gene worthy of in-depth study.

*ZSWIM7* is a recently identified gene associated with male fertility. This meiosis-specific gene is critical in homologous recombination and synapsis during germ cell development [35]. Polymorphisms in *ZSWIM7* may affect its expression or function, potentially disrupting meiotic progression and impairing fertility [36]. Studies have shown that Genetic variations in *ZSWIM7* are associated with altered reproductive phenotypes, including gametogenic defects [37]. For example, a recurrent *ZSWIM7* mutation (c.231_232del) leads to decreased meiotic recombination and male infertility in humans (*Homo sapiens*) [36]. The *ZSWIM7* c.173C > G mutation results in a loss-of-function of p (Ser58*), possibly contributing to human infertility [35]. Similar results have been reported for mice (*Mus musculus*) [38], pigs (*Sus Scrofa*) [39], and Atlantic salmon (*Salmo salar*) [40]. The present results showed that the SNP3 (c.23T/C) genotype was significantly correlated with key development indicators of the testis, such as GSI and the ratio of mature spermatozoa in 4nAT lineage. LD analysis and haplotype construction also supported a significant correlation between the diplotypes of the *ZSWIM7* gene and GSI 4nAT lineage. These results indicated that SNP3 (c.23T/C) is a definitive marker of reproductive development and could be used in practical breeding programs in the future.

Owing to the duplicated genome, identifying an effective biomarker for allotetraploids is difficult [41]. In the present study, we used genomic resequencing to scan highly variable genes and analyzed the correlations between phenotypes and genotypes. Using bioinformatics methods, we eliminated potential false-positive signals caused by genome duplication. Subsequently, we confirmed the key gene loci by analyzing the association between phenotype and genotype. These two complementary strategies ensured the accuracy of the final genotype–phenotype associations.

Having established a correlation between *ZSWIM7* genotypes and testicular development, we investigated its tissue expression profiles in the 4nAT lineage and found high expression in testes. This finding aligns with studies in mice and humans where *ZSWIM7* is primarily expressed in germline and reproductive tissues [35], particularly during meiosis [42]. Spatial localization in the 4nAT testis revealed ZSWIM7 signals around the seminiferous tubules, particularly in the basal compartment containing spermatogonia and early spermatocytes. This indicates that *ZSWIM7* participates in the transition from spermatogonia to primary spermatocytes by functioning in meiotic initiation and early spermatogenic development, potentially through regulating DNA repair and homologous recombination in early meiosis [35,42]. Given this specific localization, *ZSWIM7* could serve as a molecular marker for early germ cells [43], and its aberrant expression may cause meiotic arrest or spermatogenic failure, ultimately leading to male infertility.

## 5. Conclusions

In this study, we identified a polymorphic site, the genotype of which is closely associated with testicular development in tetraploid fish and may serve as a molecular marker for maintaining tetraploid fertility. Moreover, we revealed for the first time that *ZSWIM7* is specifically expressed in early spermatocytes in the testes of tetraploid fish. These findings provide supporting evidence for the potential use of *ZSWIM7* as a breeding marker and offer insights into its functional role in fish reproduction. This study presents a new strategy for marker identification in polyploid animals by integrating genome resequencing with genotype-phenotype association analysis. However, a limitation of this study was its relatively small sample size. In future studies, we plan to use *ZSWIM7* as a breeding marker to propagate allotetraploid fish and maintain their fertility.

## Data Availability

All data generated or analyzed during this study are included in the manuscript and Supporting Files. The raw data of whole genome re-sequencing reported in this paper have been deposited in the Genome Sequence Archive [44] in the National Genomics Data Center [45], China National Center for Bioinformation/Beijing Institute of Genomics, Chinese Academy of Sciences (GSA: CRA036280) that are publicly accessible at https://ngdc.cncb.ac.cn/gsa (accessed on 4 January 2026).

## Supplementary Materials

The following supporting information can be downloaded at https://www.mdpi.com/article/10.3390/ani16020352/s1, Figure S1: An automated system for morphological measurement in fish; Figure S2: SNP statistics of 4nAT genomic resequencing based on the reference genome of goldfish (ploidy = 2); Figure S3: SNP statistics of 4nAT genomic resequencing based on the reference genome of goldfish (ploidy = 4); Figure S4: SNP statistics of 4nAT genomic resequencing based on the combined reference genome of goldfish and common carp (ploidy = 2); Figure S5: Identification of high-frequency mutation regions in the 4nAT genome by FST and XP-CLR analysis based on the reference genome of goldfish (ploidy = 4); Figure S6: Identification of high-frequency mutation regions in the 4nAT genome by FST and XP-CLR analysis based on the reference genome of goldfish (ploidy = 2); Table S1: Morphological feature measurements of thirty male 4nAT individuals; Table S2: Read quality control summary after filtering for each sample; Table S3: Mapping statistics for each sample aligned to the goldfish reference genome; Table S4: Mapping statistics for each sample aligned to the combined goldfish-common carp reference genome; Table S5: Variants identified using the diploid goldfish reference genome; Table S6: Variants identified using the tetraploid goldfish reference; Table S7: Variants identified using the diploid combined goldfish-common carp reference genome; Table S8: Genome-wide FST scan results using the diploid goldfish reference genome; Table S9: Genome-wide XP-CLR scan results using the diploid goldfish reference genome; Table S10: Genome-wide FST scan results using the tetraploid goldfish reference; Table S11: Genome-wide XP-CLR scan results using the tetraploid goldfish reference; Table S12: Genome-wide FST scan results using the diploid combined goldfish-common carp reference genome; Table S13: Genome-wide XP-CLR scan results using the diploid combined goldfish-common carp reference genome; Table S14: Functional annotation summary of variants located in candidate regions detected by FST and XP-CLR analyses under the diploid goldfish genome; Table S15: Candidate genes associated with reproductive development detected by FST and XP-CLR analyses under the diploid goldfish genome; Table S16: Genes in the top 10% selective regions detected by XP-CLR analyses under the tetraploid goldfish genome; Table S17: The overlapping top 5% selective regions detected by FST and XP-CLR analyses under the diploid combined genome; Table S18: Genes in the overlapping top 5% selective regions detected by FST and XP-CLR analyses under the diploid combined genome. Table S19. Primers used in the present study; Table S20: Genotype and allele distribution of SNP loci; Table S21: Genetic variation in snps and correlational analysis with gonadal development; Table S22: Association analysis between *ZSWIM7* gene diplotypes and gonadal development.
