# Supplementary material for: Polymorphisms and Expression Characteristics of the ZSWIM7 Gene Are Associated with the Fertility of Male Allotetraploid of Red Crucian Carp × Common Carp
Source: Animals (Basel). 2026 Jan 22;16(2):352. doi: 10.3390/ani16020352 (PMC12838221; doi:10.3390/ani16020352)
Supplement: Supplementary file 1 [file animals-16-00352-s001.zip › Supplementary Figures.docx]

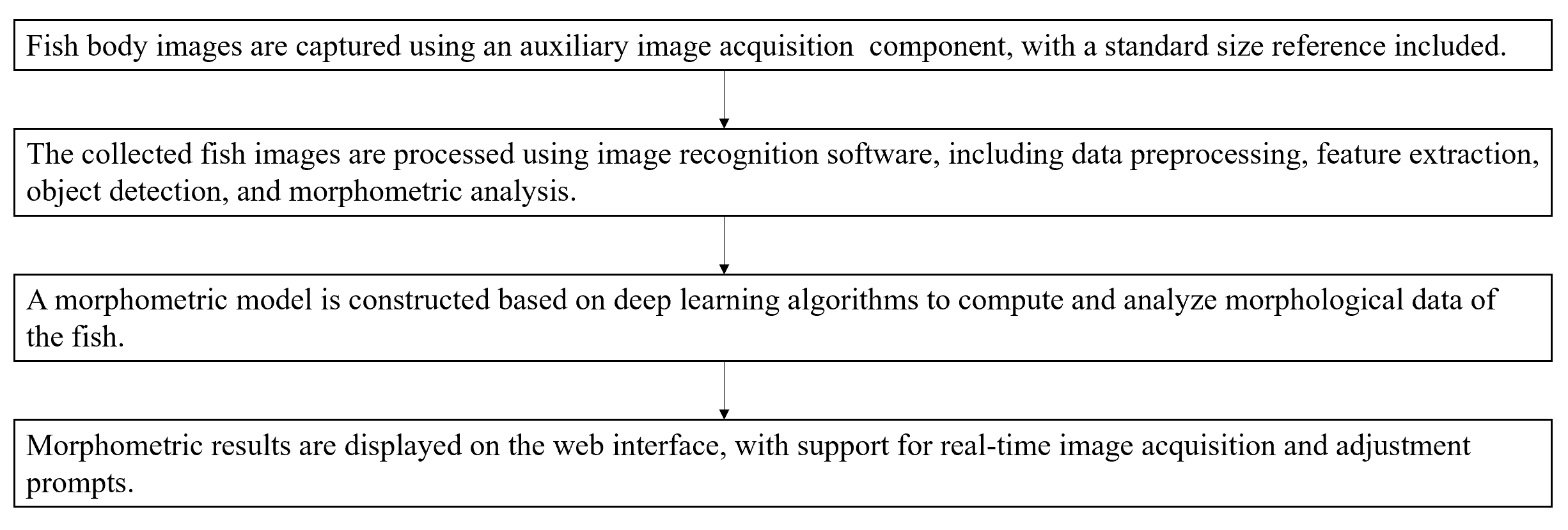


**Figure S1.** An automated system for morphological measurement in fish.


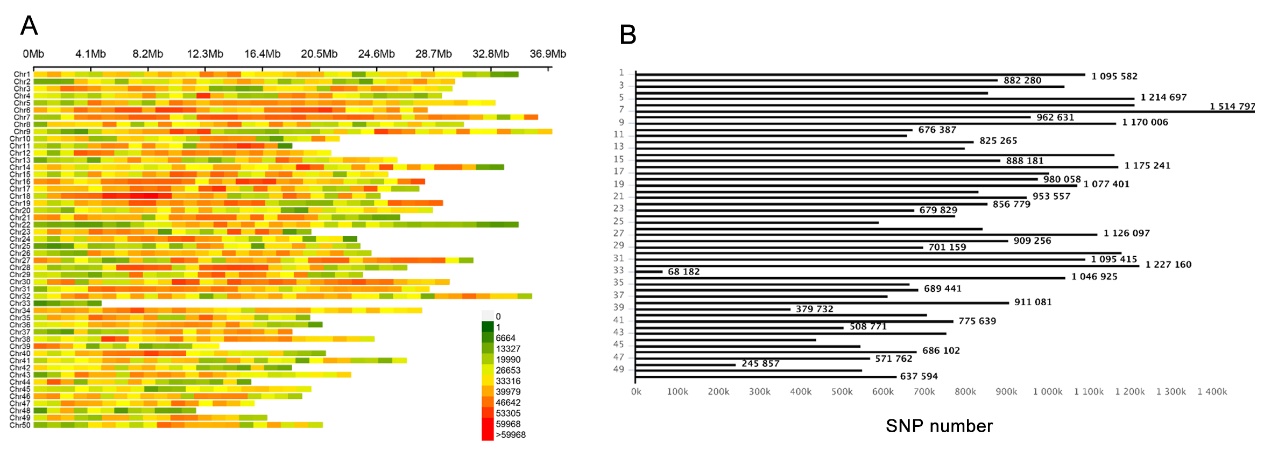


**Figure S2.** SNP statistics of 4nAT genomic resequencing based on the reference genome of goldfish (ploidy = 2). (A) The SNP density of each chromosome within 1 Mb windows size. (B) The SNP numbers of each chromosome.


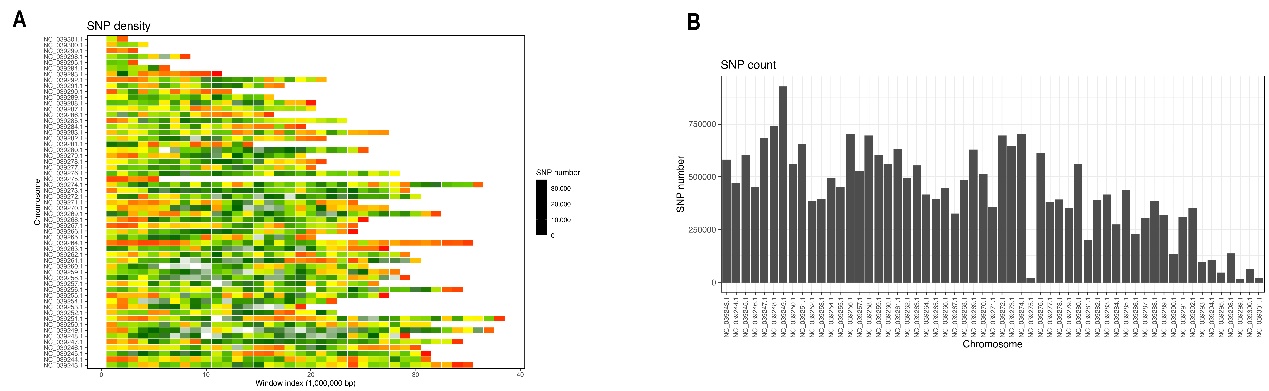


**Figure S3.** SNP statistics of 4nAT genomic resequencing based on the reference genome of goldfish (ploidy = 4). (A) The SNP density of each chromosome within 1 Mb windows size. (B) The SNP numbers of each chromosome.


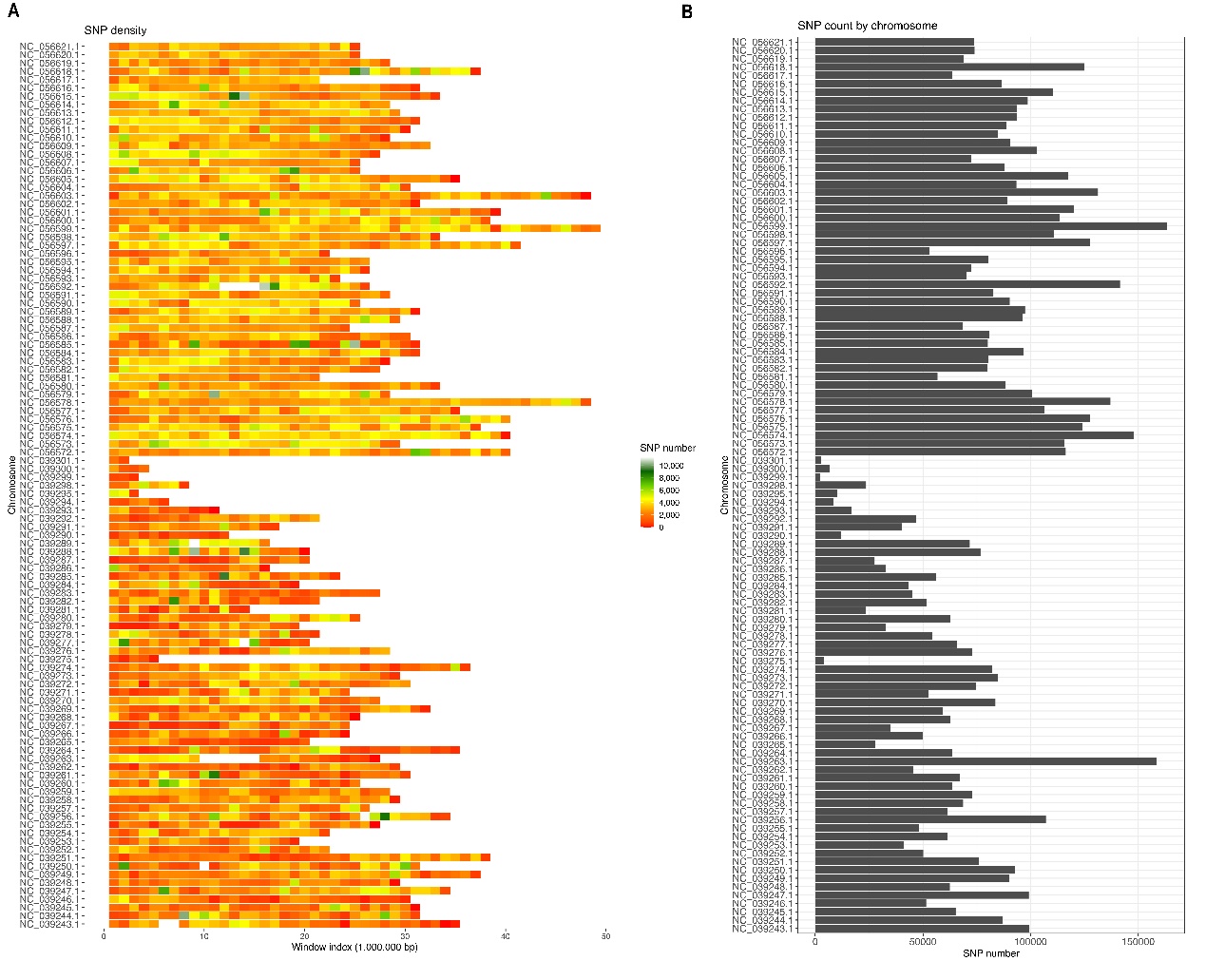


**Figure S4.** SNP statistics of 4nAT genomic resequencing based on the combined reference genome of goldfish and common carp (ploidy = 2). (A) The SNP density of each chromosome within 1 Mb windows size. (B) The SNP numbers of each chromosome.


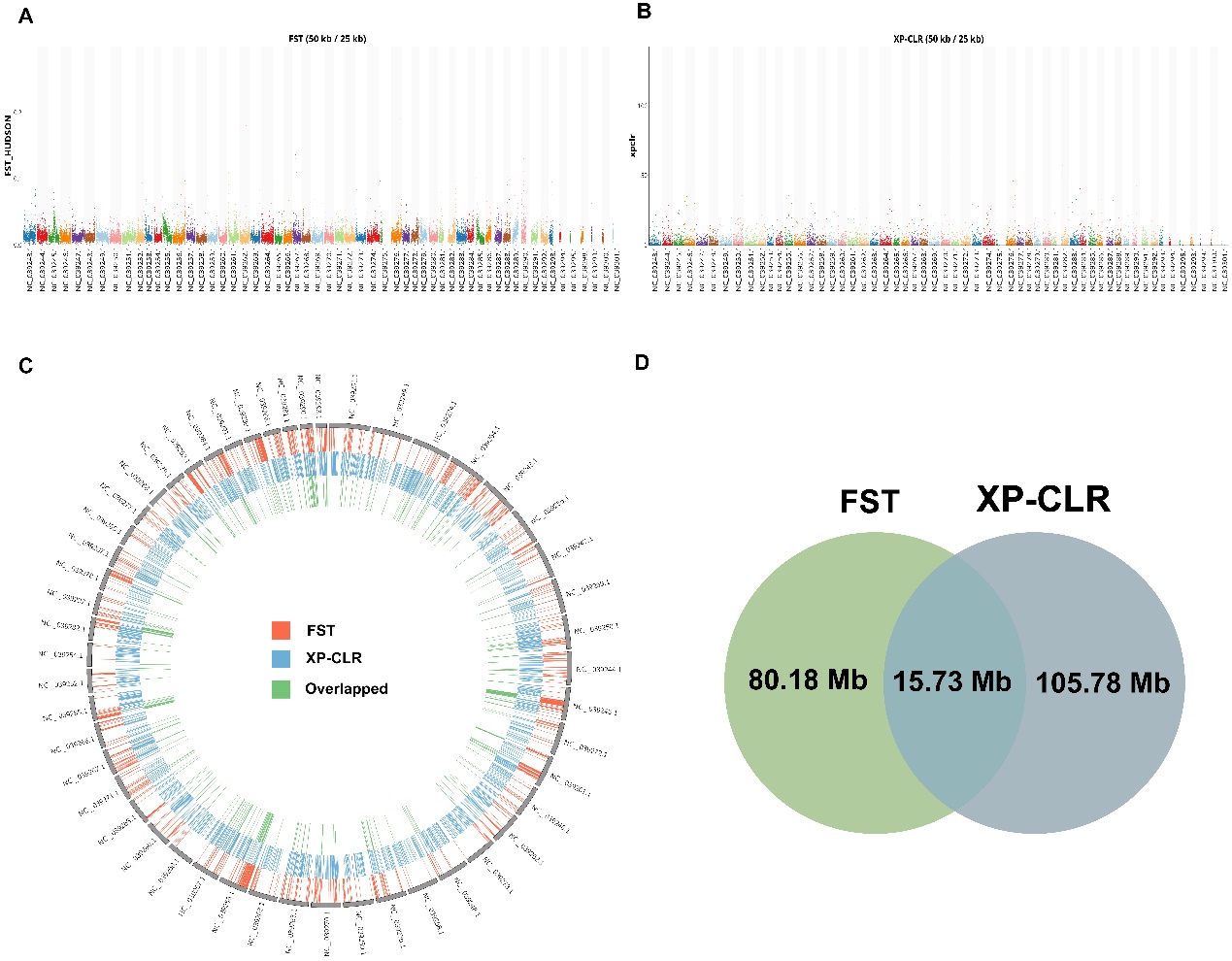


**Figure S5.** Identification of high-frequency mutation regions in the 4nAT genome by FST and XP-CLR analysis based on the reference genome of goldfish (ploidy = 4). (A) Manhattan plot of FST values (calculated in 50 kb windows with a 25 kb step size), with the y-axis representing FST and the x-axis indicating chromosomes. (B) Manhattan plot of XP-CLR scores (calculated in 50 kb windows with a 25 kb step size), with the y-axis representing XP-CLR and the x-axis indicating chromosomes. (C) Circos plot showing genomic distribution of selective regions detected independently by FST, XP-CLR, or both methods. (D) Venn diagram illustrating the overlap in selective region sizes captured by FST and XP-CLR methods.


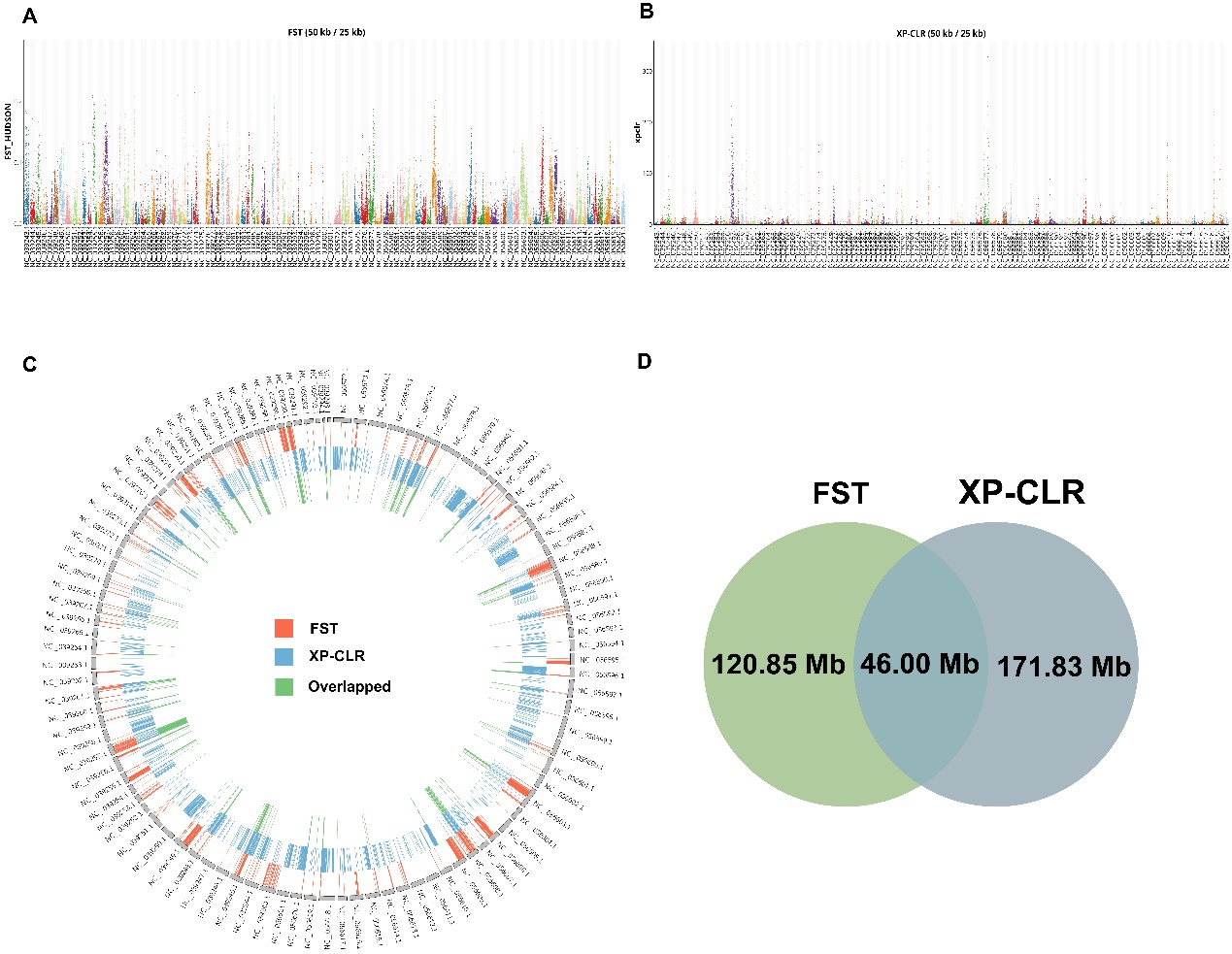


**Figure S6.** Identification of high-frequency mutation regions in the 4nAT genome by FST and XP-CLR analysis based on the reference genome of goldfish (ploidy = 2). (A) Manhattan plot of FST values (calculated in 50 kb windows with a 25 kb step size), with the y-axis representing FST and the x-axis indicating chromosomes. (B) Manhattan plot of XP-CLR scores (calculated in 50 kb windows with a 25 kb step size), with the y-axis representing XP-CLR and the x-axis indicating chromosomes. (C) Circos plot showing genomic distribution of selective regions detected independently by FST, XP-CLR, or both methods. (D) Venn diagram illustrating the overlap in selective region sizes captured by FST and XP-CLR methods.
